# An Improved LC-MS/MS Method for Simultaneous Determination of the Eleven Bioactive Constituents for Quality Control of Radix Angelicae Pubescentis and Its Related Preparations

**DOI:** 10.1155/2015/365093

**Published:** 2015-05-20

**Authors:** Jin Li, Qiu-Hong Zhang, Jun He, Er-wei Liu, Xiu-mei Gao, Yan-xu Chang

**Affiliations:** ^1^Tianjin State Key Laboratory of Modern Chinese Medicine, Tianjin University of Traditional Chinese Medicine, Tianjin 300193, China; ^2^Tianjin Key Laboratory of Phytochemistry and Pharmaceutical Analysis, Tianjin University of Traditional Chinese Medicine, Tianjin 300193, China

## Abstract

An improved LC-MS/MS method was developed for simultaneous determination of eleven bioactive constituents of Radix Angelicae Pubescentis and its related preparations. It was the first report on the quantification of bioactive constituents in different preparations of Radix Angelicae Pubescentis by LC-MS/MS analytical method. These samples were separated with an Agilent Zorbax Extend reversed-phase C18 column (1.8 *μ*m, 4.6 × 100 mm) by linear gradient elution using aqueous ammonium acetate and acetonitrile as mobile phase. The flow rate was 0.3 mL min^−1^. The eleven bioactive constituents showed good regression (*R* > 0.990) within test ranges and the recoveries were in the range of 87.1–110%. The limit of detections and quantifications for most of the major constituents were less than 0.5 and 1.0 ng mL^−1^, respectively. All results indicated that the developed method could be readily utilized as a suitable quality control method for Radix Angelicae Pubescentis and related preparations.

## 1. Introduction

Radix Angelicae Pubescentis (RAP) is one of the most commonly used traditional medicines in China for the treatment of rheumatic diseases (Pharmacopoeia of the People's Republic of China, 2010). It was named as Duhuo in Chinese. Recent pharmacological studies have revealed that it has anti-inflammatory and analgesic activities [1, 2], antiparasitic [3, 4], and inhibitory effects on 5-lipoxygenase and cyclooxygenase [5]. Various traditional Chinese medicinal prescriptions containing RAP are commercially available, such as Duhuo Jisheng pill, Tianma capsule, Tianma pill, Shujin pill, Shufengdingtong pill, Zhonghua Dieda pill and Zhuangguguanjie pill. Most of the above of these preparations have been used in the treatment of pain and inflammatory diseases [6, 7]. The demand for RAP is still increasing and becomes more and more important. Furthermore, the composition of the various bioactive compounds in Radix Angelicae Pubescentis varies significantly with many factors such as climate conditions, geographic locations, development stages, and harvest time [8, 9]. Due to the existence of such differences, the quality of Radix Angelicae Pubescentis and its related preparations also varied greatly. Therefore, it was very necessary to establish the method for control of the quality of RAP and related preparations.

A number of studies have been performed on the isolation and identification of the constituents of this herbal medicine. To date, more than 60 coumarins were isolated and identified from this plant [10–12]. It was already demonstrated that coumarins were putative active components. For example, HPLC-based activity profiling of the active extract revealed five coumarins (columbianetin acetate, imperatorin, osthol, and columbianadin) responsible for the GABAA receptor modulating activities [13]. It was reported that the angelol-type coumarins showed significant activity on human platelet aggregation [14]. Columbianetin has anti-inflammatory effect on activated human mast cells [15]. Osthole, isolated from RAP, has antiproliferative effect in rat vascular smooth muscle cells. It was also noticed that some coumarins have been linked to phototoxic, mutagenic, carcinogenic, and hepatotoxic effects [16, 17]. With these various functions in mind, it is not surprising that coumarins impact significantly on the clinical efficacy and safety of herbal medicine. Thus, coumarins were thought to be the most important bioactive markers for controlling quality of RAP and related prescriptions.

Although many analytical methods have been published about quality control of RAP, many questions still remained. Studies monitoring marker components have relied on HPLC-UV [18–20]. Based on the fact that the LC-MS/MS method was increasingly being used because of the additional specificity that could be obtained, an LC-MS/MS method was developed to simultaneously quantitate eleven bioactive constituents. Although three components (imperatorin, isoimperatorin, and osthole) in Peucedanum ostruthium (L.) Koch have been determined by HPLC-DAD [21] or six components (scopoletin, xanthotoxin, bergapten, imperatorin, osthole, and isoimperatorin) have been determined by LC-MS among the eleven compounds [22], the content of the other bioactive constituents is typically not determined by LC-MS/MS. This potentially important information is thus not available to the consumers.

In this paper, a multicomponent LC-MS/MS method of RAP was developed and validated. As an application, 11 batches of RAP and related preparations collected from different regions of China were analyzed using the established methods.

## 2. Experimental

### 2.1. Materials and Reagents

The RAP and related preparations samples were purchased from drugstores around Tianjin. Samples 1–4 were decoction pieces of RAP, and samples 5–11 were TCM preparations, namely, DuhuoJishengwan (20100602), Tianma capsule (20100111), Tianma pill (104417), Shujing pill (3990016), Shufengdingtong pill (100101), Zhonghuadida pill (091241), and Zhuangguguanjie pill (0810252). Umbelliferone, scopoletin, xanthotoxin, psoralen, and imperatorin were purchased from the National Institute for Control of Biological and Pharmaceutical Products of China. Columbianetin, bergapten, columbianetin acetate, osthol, isoimperatorin, and columbianadin were isolated from the roots of Angelicae pubescentis by Dr. Yanru Deng. Their structures were unambiguously confirmed according to their spectral data and their purities determined by HPLC were higher than 98%. Acetonitrile was chromatographically pure and was purchased from Dima Technology Inc. (USA). Deionized water was purified with a Milli-Q Academic ultrapure water system (Billerica, MA, USA) prior to the use as HPLC mobile phase. Methanol of HPLC grade was purchased from Tianjin Concord Science Co. Ltd. (Tianjin, China). Ammonium acetate was purchased from GuangFu Fine Chemicals Institute of Tianjin (Tianjin, China).

### 2.2. Instrumentation and Conditions

#### 2.2.1. Liquid Chromatography

All separations were carried out on an Agilent 1200 liquid chromatography system (Agilent, USA) equipped with a quaternary solvent delivery system, an autosampler, and a column compartment. The chromatographic separation was performed on a Zorbax Extend reversed-phase C_18_ column (4.6 × 100 mm I.D., 1.8 *μ*m particle size) and an Agilent Zorbax C_18_ guard column. A linear gradient elution of eluents *A* (1 mmoL/L, aqueous ammonium acetate) and *B* (acetonitrile) was used for the separation. The elution programmer was optimized and conducted as follows: initial 0–11 min, linear change from 70 : 30 (*A* :* B*, v/v) to 65 : 35 (*A* :* B*, v/v); 11–15 min, linear change to 33 : 67 (*A* :* B*, v/v); 15–35 min, linear change to 32 : 68 (*A* :* B*, v/v); and 35-36 min, linear change to 70 : 30 (*A* :* B*, v/v). Reequilibration time of gradient elution was 5 min. The flow rate was 0.3 mL/min, and the sample injection volume was 5 *μ*L.

#### 2.2.2. Mass Spectrometer

The LC-MS/MS analyses were conducted on an API 3200 system from Applied Biosystems/MDS Sciex (Applied Biosystems, Foster City, CA, USA). The instrument was operated with an electrospray ionization source running in positive modes in a single run, and the ion spray voltage was set to 4500 kV. The turbo spray temperature was maintained at 450°C. The nebulizer gas (gas 1) and heater gas (gas 2) were set at 40 and 40 arbitrary units, respectively. The curtain gas was kept at 20 arbitrary units, and the interface heater was turned on. Nitrogen was used in all cases. Multiple-reaction monitoring mode was employed for quantification. The precursor-to-product ion pair, declustering potential (DP), collision energy (CE), collision cell exit potential (CXP), and entrance potential (EP) for each analyte and digoxin are described in Table 1. The dwell time of each ion pair was 100 ms. All instrumentation was controlled and synchronized by Analyst software (version 1.4.2) from Applied Biosystems/MDS Sciex.

#### 2.2.3. Preparation of Sample Solutions

The dried decoction pieces of RAP samples were powdered to a homogeneous size by a mill. The capsule and pills were ground in a mortar. Each solid sample (60 mesh, 0.100 g) was accurately weighed and extracted with 10 mL of methanol by using an ultrasonic bath for 40 min and then cooled at room temperature. Methanol was added to compensate for the lost weight. The solution was filtered through a membrane filter (0.22 *μ*m), and an aliquot of 5 *μ*L of the filtrate was injected into LC-MS/MS for analysis.

#### 2.2.4. Method Validation

Methanol stock solutions containing the eleven bioactive constituents were prepared and diluted to appropriate concentrations for construction of the calibration curves. The eleven coumarin solutions at 6 different concentrations were injected in triplicate and the calibration curves were constructed by plotting the peak area ratios of coumarins to IS versus the concentrations of each bioactive constituent. The LOD and LOQ of bioactive constituents were investigated on the basis of the results for three replicates of coumarins mixed standard stock solution at different levels, considering a signal-to-noise ratio of 3 and 10, respectively. The precision and stability of the developed method were evaluated at three concentration levels (low, medium, and high) of mixed standard solutions. Intraday precision was validated with three concentrations of mixed standard solutions for five times within 1 day. Interday precision was validated with the mixed standard solutions used above once a day for 3 consecutive days. The inter- and intraday precisions for all investigated components were expressed as relative standard deviation (RSD). To evaluate the reproducibility of our studies, six independently prepared samples of herb, capsule (batch number 20100111), and pill (batch number 0810252) in parallel were prepared and analyzed. Variations were expressed as RSD. Stability was tested at room temperature and samples were analyzed in triplicate every 8 h within 24 h. Samples 1 (herb), 3 (Tianma capsule), and 11 (Zhuangguguanjie pill) were selected to test recoveries of preparation of sample solutions.

## 3. Results and Discussion

### 3.1. Optimization of Extraction Conditions

In order to achieve an efficient extraction of active components in RAP, key factors such as methanol concentration (50, 70, and 100%, v/v), sample-solvent ratio (1 : 25, 1 : 50, and 1 : 100, w/v), and ultrasonic time (20, 40, and 60 min) were investigated by using orthogonal L9 (3^4^) experiment. The optimum sample extraction condition was achieved by using 100 times of 70% (v/v) methanol for 20 min.

### 3.2. Optimization of Chromatographic Separation for Constituents

It is well known that choice of mobile phase and an appropriate elution program were of importance for the separation multicomponents in TCM. Based on previous studies, acetonitrile was selected as the organic phase in the mobile phase considering its better eluting power to the coumarins. A short column packed with 1.8 *μ*m particles (Eclipse plus C18 column 4.6 × 100 mm, 1.8 *μ*m) using acetonitrile-water system was effective for the separation of samples of RAP and its related preparations. Furthermore, gradient elution possessed a distinct advantage for separating these eleven coumarins and a high sensitivity for determination by using MS/MS. It was reported that electrolyte modification ammonium acetate of mobile phase could significantly improve the ESI efficiency resulting in enhanced furocoumarins (xanthotoxin, psoralen, isopimpinellin, and bergapten) responses [23]. According to our previous research [24], a solvent system consisting of acetonitrile and 1 mmol ammonium acetate aqueous solution, which could provide greater baseline stability and higher sensitivity of analyte, was ultimately selected as mobile phase system.

### 3.3. Optimization of Mass Spectra

In the present study, ESI-MS analyses of eleven bioactive constituents were performed in both the positive-ion and negative-ion modes. The results showed that positive electrospray ionization could offer better sensitivity and reproducibility. Thus, positive mode was finally selected to detect the eleven bioactive constituents.

In order to optimize the MRM assay for each bioactive constituent, standard methanol solution of eleven bioactive constituents was infused into the ESI source with a syringe pump. It was found that the ESI-MS spectra of the eleven bioactive constituents were dominated by the presence of the [M + H]^+^ deprotonate molecule in the positive mode. IS were dominated by the presence of the [M + NH_4_]^+^ deprotonate molecule in the positive mode. A variety of molecular ions for digoxin, including [M – H_2_O]^+^, [M + H]^+^, [M + NH_4_]^+^, [M + Na]^+^, and [M + K]^+^, were observed in Q1 positive full-scan with respective *m*/*z* at 763.9, 781.9, 798.9, 803.9, and 819.7, respectively. A higher abundance was found for the ammonium addition of [M + NH_4_]^+^, which was used for further fragmentation in product ion scan. The product ions were obtained by fragmentation of the ammonium adduct precursor ion in a collision cell. Multiple-reaction monitoring (MRM) mode was used for quantitative detection, with sensitive ion transitions of *m*/*z* 798.6/113.3. Thus, the [M + H]^+^ ions for those of target compounds and [M + NH_4_]^+^ for IS were selected as the precursor ions and fragmented in MS/MS mode to obtain the prominent product ions, respectively.

The quantification of the analytes and IS was performed using the MRM data acquisitions and twelve pairs of MRM transitions which were listed in Table 1 were selected. Under the optimized LC conditions, retention times of eleven coumarins and IS were obtained from multiple injections of samples during the entire analysis time period. The retention times of scopoletin, umbelliferone, columbianetin, psoralen, xanthotoxin, bergapten, columbianetin acetate, imperatorin, osthol, isoimperatorin, and columbianadin were 7.72, 8.52, 10.42, 14.29, 19.81, 20.21, 21.30, 23.06, 27.84, 29.96, 31.48 32.84 min, respectively. Certain tendencies in the elution order were as follows: scopoletin, umbelliferone, columbianetin, psoralen, xanthotoxin, bergapten, columbianetin acetate, imperatorin, osthol, isoimperatorin, and columbianadin. From Figures 2 and 3, it could be seen that there were no interference peaks at the retention positions of the analytes and IS to interfere with the analysis of samples.

### 3.4. Selection of Internal Standard

Although the molecular weight and chemical structure of digoxin are much different from those of target compounds, digoxin is easily obtained from market and has good sensitivity in the positive mode and good separation from the target compounds in the chromatogram. Therefore, digoxin is selected as internal standard in this study to calculate multicomponents.

### 3.5. Method Validation

The detailed descriptions of the regression curves were shown in Table 2. The good linearities (coefficient of determination *R* ^2^ > 0.990) were obtained in test ranges for all bioactive compounds. The values of LOD and LOQ of bioactive constituents were shown in Table 2. The LODs of all investigated bioactive constituents except for umbelliferone were below 1.5 ng mL^−1^ in the established method. The results of reproducibility and stability tests were shown in Table 3. The results of reproducibility tests are shown in Table 4. Table 4 also illustrated the data of recovery obtained in Radix Angelicae Pubescentis and its related preparations. The average recoveries of the eleven investigated compounds were 87.3–110% and their RSD values were lower than 10%. It was concluded that the proposed HPLC-MS/MS method was precise, accurate, and sensitive enough for simultaneous quantitative evaluation of the eleven coumarins in samples of RAP and its related preparations.

### 3.6. Application to Real Samples

**Figure 1:**
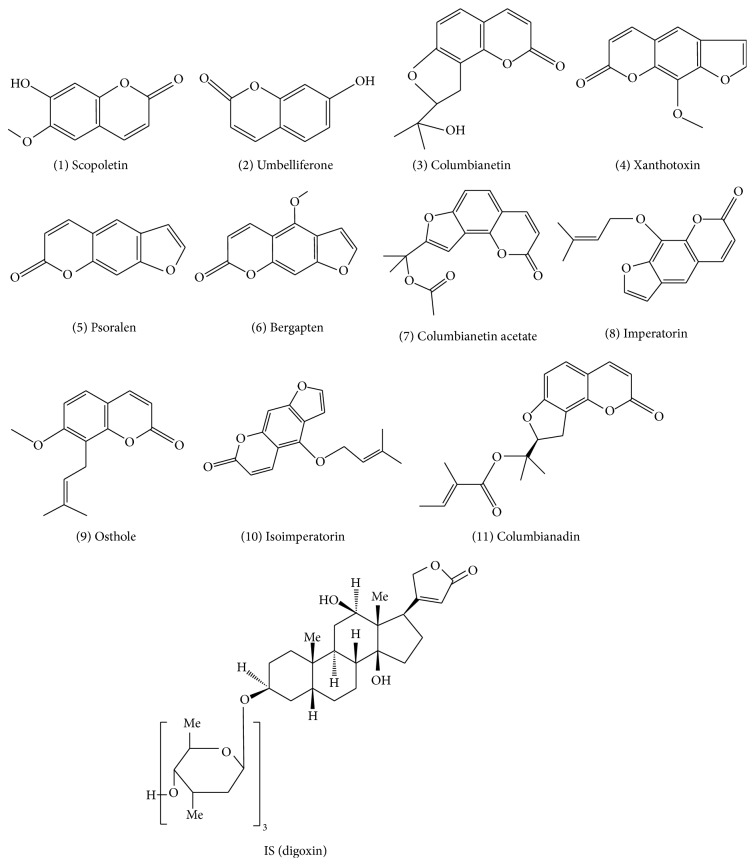
Chemical structures of eleven major active constituents.

**Figure 2:**
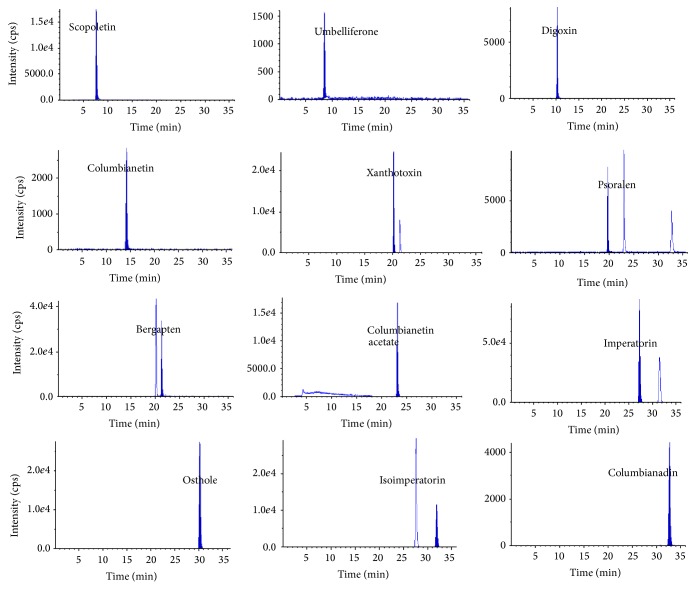
Typical MRM chromatograms of eleven authentic standards Mixed.

**Figure 3:**
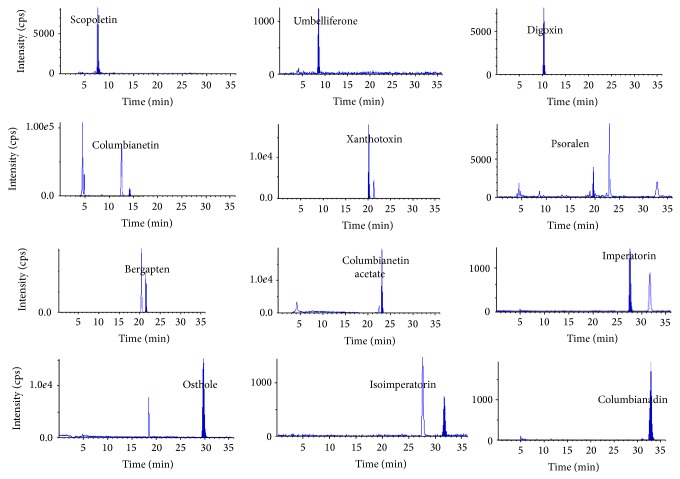
Typical MRM of Radix Angelicae Pubescentis.

The developed LC-MS/MS method was subsequently applied for the analysis of eleven major constituents in eleven batches of commercial samples of RAP and its related preparations (Figure 1). These samples include four RAP, one capsule, and six pills samples. The typical MRM chromatograms of authentic standards Mixed and RAP which were obtained were shown in Figures 2 and 3, respectively. The results of the contents of eleven major constituents in samples of Radix Angelicae Pubescentis and its related preparations were listed in Table 5. The results showed that the contents of eleven major constituents in their related preparations were distinct from those in Radix Angelicae Pubescentis. PPRC 2010 edition established the quality control standards for Radix Angelicae Pubescentis as follows: the osthol and columbianadin contents should not be less than 5, 0.8 mg/g, respectively. The marker compounds contents in these 4 batches of commercial RAP all did not exceed the minimum standards of PPRC. Comparing the contents of active components in preparation with that in raw herbs, most of contents of the active component in related preparations were less than those in RAP except psoralen in the Zhuangguguanjie pills, because psoralen comes from Buguzhi which was another TCM contained in Zuangguguanjie pills. These results suggested that the developed method could be used to evaluate the quality control for this important Chinese herbal medicine and its related preparations.

## 4. Conclusion

The proposed method made it possible to determine simultaneously the eleven main bioactive components in samples of RAP and its related preparations in one run with acceptable levels of linearity, precision, repeatability, and accuracy. It was the first report to reveal the distribution of eleven chemical constitutes in RAP and its related preparations by the LC-MS/MS analytical method. The results demonstrated that the developed method could be applied as a reliable and sensitive quality control for this important Chinese herbal medicine and its related preparations.

## Figures and Tables

**Table 1:** Retention time, MS/MS fragment ions, declustering potential (DP), and collision energy (CE) of the 11 coumarins compounds in Radix Angelicae Pubescentis.

Compounds	Q1	Q3	DP (V)	EP (V)	CEP (V)	CE (V)	CXP (V)
Scopoletin	193.2	133.1	42	3	20	29	3.9
Umbelliferone	163.1	107.3	38	2	11	28	2
IS (digoxin)	798.6	113.3	25	3.8	35	68	2
Columbianetin	246.8	175.1	61	9	16.8	29	3
Psoralen	187.2	131.1	42	9	20	29	10
Bergapten	217.4	202.3	44	7	15.8	30	3
Xanthotoxin	217.4	174.1	46	8.8	11	34	16
Columbianetin acetate	288.9	229.1	46	2.9	18	12	4
Imperatorin	271.3	203.1	49	10	31	15	17
Osthole	244.8	189.2	53.8	3.7	16.8	20	4
Isoimperatorin	271.3	203.1	29	2	17.6	17	3
Columbianadin	329.4	229.2	42	3	19.5	32	2

**Table 2:** The calibration curves, LODs and LOQs (*n* = 5) for the active components of the assay.

Compounds	Regression equation^(a)^	*r*	Linear range	LOD^(b)^	LOQ^(c)^
			(ng mL^−1^)	(ng mL^−1^)	(ng mL^−1^)
Scopoletin	*Y* = 0.0175*X* + 0.0403	0.9995	1–500	0.30	1.0
Umbelliferone	*Y* = 0.00219*X* + 2.69*E* − 015	0.9992	10–1000	0.30	1.0
Columbianetin	*Y* = 0.00373*X* + 0.0112	0.9990	5–1000	0.50	1.7
Psoralen	*Y* = 0.00634*X* + 0.0025	0.9990	10–5000	0.33	1.0
Bergapten	*Y* = 0.0309*X* + 0.259	0.9999	1.1–1100	0.30	1.0
Xanthotoxin	*Y* = 0.0189*X* + 0.0198	0.9990	10.7–1070	0.50	1.5
Columbianetin acetate	*Y* = 0.0152*X* + 0.0103	0.9992	13–1300	0.30	1.0
Imperatorin	*Y* = 0.0237*X* + 0.145	0.9998	5–1000	0.02	0.2
Osthole	*Y* = 0.00395 + 0.00386	0.9998	12.7–1270	0.01	0.1
Isoimperatorin	*Y* = 0.123*X* + 0.0497	0.9999	10.3–1030	0.01	0.1
Columbianadin	*Y* = 0.00659*X* + 0.00819	0.9990	13–1300	0.17	0.5

^(a)^In the regression equation, the *X* value is the concentration of compounds (ng·mL^−1^), and the *Y* value is the peak area.

^(b)^Limit of detection.

^(c)^Limit of quantification.

**Table 3:** Intra-assay and interassay precision of the developed method (*n* = 6).

Components	Concentration	Intra-assay	Interassay	Stability
	(ng mL^−1^)	RSD (%)	RSD (%)	RSD (%)
Scopoletin	20.6	5.03	6.17	9.46
	103	3.16	5.20	5.44
	1030	2.13	4.91	5.37

Umbelliferone	20	9.04	9.00	9.53
	100	4.89	3.04	6.48
	1000	5.14	7.11	9.35

Columbianetin	22	7.70	9.7	5.74
	110	9.14	8.77	5.23
	1100	2.93	8.62	8.47

Psoralen	21.6	3.61	9.11	8.05
	108	5.28	9.07	6.40
	1080	2.26	7.18	7.33

Bergapten	22	3.15	7.79	6.38
	110	5.57	9.51	7.53
	1100	3.38	8.35	7.45

Xanthotoxin	21.4	6.81	6.88	5.21
	107	3.27	3.04	4.97
	1070	2.87	8.31	7.88

Columbianetin acetate	26	8.05	3.24	9.04
	130	7.84	7.59	6.05
	1300	2.77	6.80	9.01

Imperatorin	20.6	4.32	2.42	5.62
	103	8.48	3.78	6.08
	1030	3.08	7.88	8.52

Osthole	25.4	5.48	6.77	8.08
	127	3.72	5.49	2.75
	1270	3.54	4.70	5.28

Isoimperatorin	20	4.17	3.81	5.19
	100	2.98	7.05	6.68
	1000	1.94	5.67	8.23

Columbianadin	26	9.28	8.56	9.73
	130	2.90	1.45	5.84
	1300	2.98	3.14	3.25

**Table 4:** Repeatability and recovery of the developed method (*n* = 6).

Compounds	Reproducibility			Recovery^(a)^					
	Herbal	Capsule	Pill	Herbal		Capsule		Pill	
	RSD (%)	RSD (%)	RSD (%)	Mean	RSD (%)	Mean	RSD (%)	Mean	RSD (%)
Scopoletin	4.97	6.32	6.72	96.1	5.74	105	1.85	101.	0.62
Umbelliferone	8.21	8.30	5.79	106	3.38	95.8	5.49	97.9	6.90
Columbianetin	4.07	9.53	5.31	110	4.09	99.3	2.69	102	0.59
Psoralen	4.71	7.86	3.81	99.8	5.66	97.2	6.44	105	8.46
Bergapten	4.09	6.41	4.56	106	5.67	108	0.47	101	6.95
Xanthotoxin	3.83	6.64	4.57	105	1.15	106	2.13	100	2.19
Columbianetin acetate	5.77	8.88	2.05	87.1	3.87	105	5.14	106	6.04
Imperatorin	3.72	9.12	3.69	107	1.87	104	2.60	105	3.82
Osthole	5.07	7.30	4.45	106	7.71	90.3	3.62	100	8.11
Isoimperatorin	5.10	6.48	7.16	106	1.64	107	0.32	105	3.28
Columbianadin	4.75	6.64	3.82	89.9	7.31	96.4	9.09	100	5.91

^(a)^Recovery (%) = (Amount determined − Amount original)/Amount spiked × 100%.

**Table 5:** Results for the determination of the 11 components in sample (mg g^−1^).

Sample	Scopoletin	Umbelliferone	Columbianetin	Psoralen	Bergapten	Xanthotoxin	Columbianetin acetate	Imperatorin	Osthole	Isoimperatorin	Columbianadin
S1	Tr	0.0200	0.1342	0.0546	0.0647	0.1140	0.7756	0.1353	3.8504	0.0513	0.4829
S2	0.0034	0.0210	0.1199	0.0829	0.1430	0.5377	0.7525	0.1069	3.8064	0.0545	0.6304
S3	0.0040	0.05810	0.1822	0.0489	0.0766	0.2560	0.6022	0.0479	2.6000	0.0403	0.6289
S4	Tr	0.07020	0.1232	0.0484	0.0320	0.0510	0.6865	0.0543	2.3652	0.0127	0.6249
S5	0.0408	Tr	0.0269	0.1756	0.0021	0.04	Tr	Tr	Tr	0.006	0.0084
S6	0.0011	Tr	0.0142	0.0056	0.0043	0.0005	0.0006	0.02	0.0030	0.0009	0.0038
S7	0.0019	0.0069	0.0194	0.0018	0.0024	0.0020	0.0135	0.0537	0.0720	0.0030	0.0385
S8	Tr	0.0062	0.0046	0.0008	Tr	0.0010	0.0026	0.0038	0.0125	0.0006	0.0075
S9	Tr	Tr	0.0013	0.0014	Tr	0.0006	0.0024	Tr	0.0153	0.0002	0.0053
S10	0.0004	Tr	0.0170	0.0023	0.0006	0.0020	0.0047	Tr	0.0374	0.0002	0.0067
S11	0.0003	0.0028	0.0173	0.2475	0.0012	0.0040	0.0117	0.0004	0.0413	0.0006	0.1052

Tr: Trace.
